# Correlated order at the tipping point in the kagome metal CsV_3_Sb_5_

**DOI:** 10.1038/s41567-023-02374-z

**Published:** 2024-01-31

**Authors:** Chunyu Guo, Glenn Wagner, Carsten Putzke, Dong Chen, Kaize Wang, Ling Zhang, Martin Gutierrez-Amigo, Ion Errea, Maia G. Vergniory, Claudia Felser, Mark H. Fischer, Titus Neupert, Philip J. W. Moll

**Affiliations:** 1 Max Planck Institute for the Structure and Dynamics of Matter, Hamburg, Germany; 2 Department of Physics, University of Zürich, Zürich, Switzerland; 3 Max Planck Institute for Chemical Physics of Solids, Dresden, Germany; 4 College of Physics, Qingdao University, Qingdao, China; 5 Centro de Física de Materiales (CSIC-UPV/EHU), Donostia-San Sebastian, Spain; 6 Department of Physics, University of the Basque Country (UPV/EHU), Bilbao, Spain; 7 Donostia International Physics Center, Donostia-San Sebastian, Spain; 8 Fisika Aplikatua Saila, Gipuzkoako Ingeniaritza Eskola, University of the Basque Country (UPV/EHU), Donostia-San Sebastian, Spain

**Keywords:** Electronic properties and materials, Topological defects

## Abstract

Spontaneously broken symmetries are at the heart of many phenomena of quantum matter and physics more generally. However, determining the exact symmetries that are broken can be challenging due to imperfections such as strain, in particular when multiple electronic orders are competing. This is exemplified by charge order in some kagome systems, where evidence of nematicity and flux order from orbital currents remains inconclusive due to contradictory measurements. Here we clarify this controversy by fabricating highly symmetric samples of a member of this family, CsV_3_Sb_5_, and measuring their transport properties. We find that a measurable anisotropy is absent at any temperature in the unperturbed material. However, a pronounced in-plane transport anisotropy appears when either weak magnetic fields or strains are present. A symmetry analysis indicates that a perpendicular magnetic field can indeed lead to in-plane anisotropy by inducing a flux order coexisting with more conventional bond order. Our results provide a unifying picture for the controversial charge order in kagome metals and highlight the need for materials control at the microscopic scale in the identification of broken symmetries.

## Abstract (web summary)

The electronic transport properties of charge-ordered kagome metals are controversial. Now careful measurements on unperturbed samples show that previously measured anisotropy in the transport occurs only when external perturbations are present.

## Main

Materials hosting intertwined electronic ordering phenomena provide both an outstanding challenge and opportunity in current condensed matter physics. Phases such as magnetism, charge order, spin textures or superconductivity may cooperate or compete, or merely coexist without much commonality, thus complicating the interpretation of experimental data^1,2^. Disentangling the various order parameters into the most elemental building blocks, separating the primary from secondary orders and understanding their interrelation, is the key to solving their rich puzzle. Complex quantum materials with entangled correlated phases also offer unique electronic response functions: when manipulating one order, one can switch or tune a different one, akin to the unique electromagnetic responses of multiferroics^3,4^.

With the poor performance of ab-initio predictions for such correlated materials, a promising experimental route is to follow structural motifs that either host highly degenerate states or exhibit geometrically frustrated bonds, which are known to commonly host correlated phases at low temperatures. Honeycomb lattices, square nets, perovskite cages or the kagome lattice have been extremely fruitful in this regard^4–8^. The kagome lattice, a net of triangles connected at their vertices, combines both bond frustration and sublattice symmetries and thus has been a successful platform for the design of non-trivial quantum materials^9–16^.

Recently, the kagome family (Cs,K,Rb)V_3_Sb_5_ has received significant attention due to the wealth of phases it hosts^16–20^. At high temperature, these V-based kagome systems are in a centrosymmetric, non-magnetic metallic state, but undergo a charge-density-wave (CDW)-type instability at *T*_CDW_ ≈ 100 K. Consensus of a 2 × 2 reconstruction in the kagome plane has been reached, yet the exact low-temperature structure and the nature of the out-of-plane reconstruction (2 × 2 × 2 versus 2 × 2 × 4) remain to be clarified^21–23^. At even lower temperatures, superconductivity appears at a critical temperature *T*_c_ ≈ 2.5 K which is enhanced by hydrostatic pressure, anti-correlated with the pressure dependence of the CDW indicating their competitive relationship^24–26^. Beyond this, an impressive set of experiments has demonstrated that an additional phenomenon is happening in this material, often associated with an onset temperature of ${T}^{{\prime} }\approx 20-50{\; }\text{K}$, yet the nature and physical influences are under heavy debate, fuelled by openly contradictory experimental results. These experiments include signatures of time-reversal symmetry (TRS) breaking^27–31^ and absence thereof^32,33^; electronic nematicity^34–38^ and absence thereof^39,40^; and tuneable chirality^41–43^ and absence thereof^44^.

The central question is how such carefully conducted experiments on a deceptively simple, stoichiometric material of high crystalline purity yield such contradictory results. Here, we propose, and experimentally demonstrate, that these discrepancies are intrinsically rooted in the strong coupling of the various orders it hosts. This renders this material class extraordinarily sensitive to even weak perturbations, which could safely be ignored in conventional compounds. We demonstrate that in-plane strain and magnetic fields are such sensitive perturbations, yet the material may be highly sensitive to others as well. In the experimental reality, weakest and inhomogeneous residual strains (for example, from crystal defects and sample mounting) are ubiquitous and hard to avoid, while magnetic fields of several Tesla are used in the spirit of non-invasive probes (quantum oscillations, nuclear magnetic resonance, magnetotransport). We propose that shielding crystalline samples from perturbations as much as possible will consolidate the field and lead towards the identification of the correlated ground state without perturbations. The main message of this paper is that the at-first-glance contradictory state of the literature is a feature, not a bug. (Cs,K,Rb)V_3_Sb_5_ may well realize the long-standing dream of unusual electronic response functions that directly arise from the near-degeneracy of multiple distinct correlated states.

## Isotropic in-plane transport in zero field

**Fig. 1:**
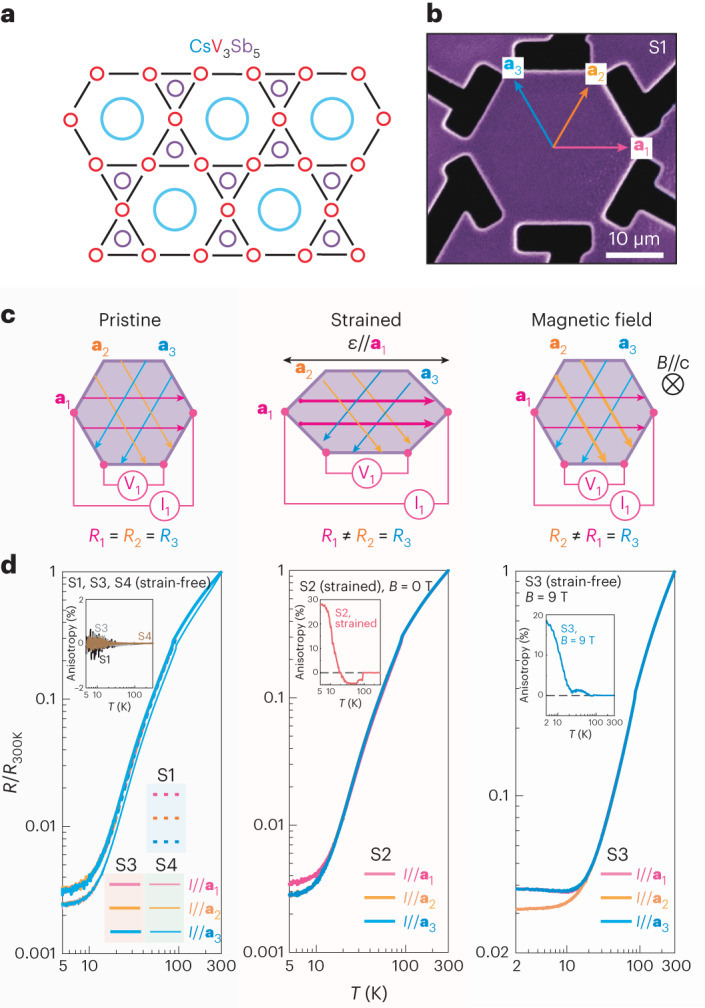
Field- and strain-induced in-plane transport anisotropy in CsV_3_Sb_5_. **a**, Detailed setup of tri-directional resistance measurement and possible origin of in-plane anisotropy. **b**, Scanning electron microscopy (SEM) image of device S1. The hexagon of CsV_3_Sb_5_ is fabricated via focused-ion-beam technique with six symmetric contacts. The three equivalent a-directions of the Kagome plane are noted as a_1_, a_2_ and a_3_, respectively. **c**, Illustration of in-plane electric transport under different conditions. With the application of in-plane strain or out-of-plane magnetic field, the resistances measured along different directions, *R*_i_ (i = 1, 2, 3), become anisotropic. Here, *I* and *V* denote the applied current and the measured voltage, respectively. **d**, Temperature (*T*) dependence of tri-directional resistance for the nearly strain-free devices S1, S3 and S4 in zero field, device S2 under strain and device S3 in the presence of magnetic field (*B* = 9 T). Source data

One proposed electronic instability is a spontaneous breaking of the six-fold rotational symmetry of the kagome plane into a two-fold symmetric electronic state^34,35,37^. Such nematic transitions manifest in an emergent transport anisotropy within the plane, which is symmetry-forbidden in a six-fold symmetric kagome plane. To this end, we have machined hexagon-shaped microstructures featuring six contacts, one at each corner of the hexagon, from bulk single crystals using focused-ion-beam (FIB) milling. Great care was taken to align the structure with the in-plane lattice vectors via X-ray diffraction (less than 0.5°) and to minimize shape deviations to avoid any symmetry lowering due to the structure’s shape itself (Fig. 1). Systematic resistance measurements were performed with current applied diagonally across the hexagon and voltage measured along the side, in all three possible configurations. As we will show even weak strains to be critical factors, it is important to remove any residual strain onto the micro-shaped crystal that arises from differential thermal contraction.

Low-strain samples have been achieved in two ways, either by decoupling the structure from the substrate by suspending it freely on ultra-soft SiN_*x*_ membranes (samples S1 and S4); or by encasing it in an epoxy droplet crafted in such a way that the compressive forces of the contracting soft glue nearly compensate the tensile forces of a hard substrate coupling (sample S3). Both low-stress designs result in an approximately strain-free hexagon at cryogenic temperatures (see Methods section for residual strain estimates and fabrication details)^25^. While in the membrane-based samples the residual strain is minimized by explicit elastic design^42,45^, their fabrication is complex. The epoxy-based method is inherently less controlled yet has proven to be quite effective in mitigating undesired strain asymmetries. This low-tech approach may also be useful in other techniques, such as scattering methods. To explicitly demonstrate the role of strain, a sample on a purposely mismatched substrate was exposed to strain via differential thermal contraction^46,47^. The effective forces acting on the structure closely resemble a force load pulling on two opposite corners of the otherwise free hexagon. The resulting microscopic strain profile in the hexagon is highly inhomogeneous yet preserves the mirror symmetry along the force direction corroborated by finite element simulations (Methods). The main aim of this strain field is to subject the sample to a weak and complex strain field as it may accidentally arise in various experimental circumstances. Sharp transitions at *T*_CDW_ and *T*_c_, in perfect agreement with the bulk values, signal the approximately strain-free state of S1, S3 and S4. Our strained sample S2 exhibits broader transitions at a shifted *T*_CDW_ and *T*_c_, in agreement with their known strain dependence and the broadening due to the strain inhomogeneity^48^ (Methods). Our set of samples allows us to probe microscopic signatures of rotational symmetry breaking with a high level of control over the physical state of the sample.

For temperatures above *T*_CDW_, the resistances measured along three current directions are identical in each of the three samples (Fig. 1), as expected for a six-fold symmetric kagome metal. Interestingly, they remain identical for the low-strain samples S1, S3 and S4 at all temperatures down to *T*_c_ within ± 0.05%. In light of the various contradictory results on the spontaneous symmetry breaking of the electronic structure in CsV_3_Sb_5_, we emphasize the argumentative power of observing the absence of anisotropy. Electronic anisotropy, if observed, may appear due to intrinsic or extrinsic symmetry breaking, yet its absence constrains us to two possible microscopic scenarios of the underlying electronic state: Either the sample decays into perfectly balanced domain structures as to restore the apparent C6 symmetry of macroscopically averaging probes such as transport; or it remains fundamentally isotropic in the absence of perturbations. The reproducibility of these results in combination with the 10–20 μm size of the hexagon necessitates extremely small domains in the nm-range, as to obtain reliably perfect averaging. Both the high density of energetically costly domain boundaries and the absence of such vastly textured electronic matter in local-probe experiments^21,30^ speak against the nano-domain picture, while we note it cannot be excluded from our data.

Importantly, we here discuss only the symmetry of the electronic fluid that governs the transport properties. There is an active debate about the crystalline symmetry describing the accurate ionic positions within the CDW state. The proposed low-temperature structures are mostly orthorhombic, which formally breaks the C6 symmetry by two main mechanisms: (1) subtle deviations of bond lengths break C6 within each plane^49^; or (2) stacking C6 symmetric layers with interlayer phase shifts reduces the global symmetry of the stack to C2 (ref. ^50^). The detailed ionic structure is highly important as it reflects the energetics of the reconstructed state over its entire band width. However, our transport study is only sensitive to the details at the chemical potential. The fact that sufficiently decoupled samples show isotropic in-plane responses does not exclude any such structural symmetry breaking effects, it merely evidences that their impact on transport is negligible. This may not be surprising as the structural distortions are so subtle that no consensus on the structure has been reached even with high-resolution X-ray studies.

A different picture appears in the strained case: At *T*_CDW_, a clearly observable resistivity anisotropy immediately appears. Two directions remain identical within experimental accuracy, while the one along the unidirectional strain direction differs. This is a direct consequence of engineering a complex strain field that remains mirror symmetric along and perpendicular to the applied force. We quantify the experimental anisotropy as the anti-symmetric difference between these two directions, (*R*_1_ − *R*_2_)/(*R*_1_ + *R*_2_). This anisotropy remains relatively featureless until it distinctly grows around ${T}^{{\prime} }\approx 30{\; }\text{K}$ to above 30% at *T* = 5 K. This sudden growth in the strained sample S2 is accompanied by a sign change of the anisotropy, occurring simultaneously with the various anomalies reported around ${T}^{{\prime} }$. These observations are fully consistent with two very recent elastoresisance works on uniaxial strain (cite) that support the non-nematic picture. Using uniaxial strain, a symmetry decomposition revealed a weak response in the nematic channel (E_2g_) yet observed a massive enhancement of the symmetric (A_1g_) response as the samples are cooled through ${T}^{{\prime} }$. This suggests the divergent anisotropy under strain likely arises from the strain gradients acting on the A_1g_ channel. The remarkable non-nematic impact of weak strain fields may be associated with the energetic stability of various close-by structures, such as the orthorhombic ones discussed above. High-precision X-ray studies under uniaxial strain are needed to clarify this point.

## Field- and strain-induced anisotropy

A second, key piece to the puzzle is unveiled by magnetic fields. Previous reports^41^ suggested that the rotational symmetry breaking is linked to time-reversal symmetry breaking. Under a static out-of-plane magnetic field *B*_c_, even strain-free samples exhibit a transport anisotropy, which increases monotonically with increasing magnetic field (Extended Data Fig. 6). For *B*_c_ = 9 T, the anisotropy onsets at *T* ≈ 70 K and reaches 20% at low temperature. Similar to the strained samples, the anisotropy increases markedly around ${T}^{{\prime} }\approx$ 30 K. Detailed rotation studies of the magnetic field away from the *c* direction have shown no influence, hence we can experimentally exclude a small accidental in-plane field component to be the source of the symmetry breaking (Extended Data Fig. 9). Repeated cooldowns exactly reproduce the state of the sample, speaking against spontaneous symmetry breaking and likely the weak but non-zero residual strain breaks the symmetry statically. Still, in the same sample without a magnetic field no anisotropy is detectable. This behaviour marks out-of-plane magnetic fields *B*_c_ as another crucial tuning parameter for electronic symmetry breaking in CsV_3_Sb_5_. The state of broken rotational symmetry can be directly controlled by a magnetic field, evidencing the strong coupling between these phenomena. Together, these observations substantiate a consistent experimental picture of an isotropic state in the pristine material that is critically unstable against forming a nematic state under weak perturbations. Indeed, when strain and out-of-plane fields are simultaneously applied, the anisotropy grows even further (Extended Data Fig. 8). Interestingly, high-resolution X-ray studies have not found any evidence for a change in the lattice structure of the CDW state under field^50^, which combined with our results further supports an unconventional type of electronic order.

## Theoretical model of symmetry analysis and strain-field coupling

**Fig. 2:**
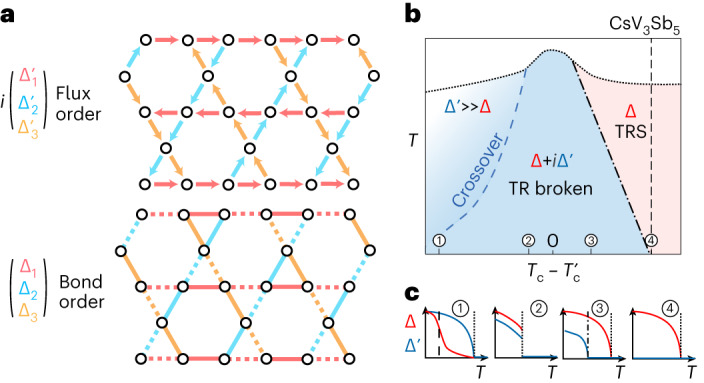
Ginzburg–Landau phase diagram. **a**, Definition of the three-component order parameters ${{{\Delta }}}$ (describing real modulation of the bond hopping) and ${{{\Delta }}}^{{\prime} }$ (describing imaginary modulation of the bond hopping, meaning flux order). Both order parameters break translational symmetry and increase the unit cell by 2 × 2. In the lower panel, solid and dashed lines denote positive and negative values of the bond order parameter, respectively. **b**, Schematic phase diagram of the order parameters ${{{\Delta }}}$ and ${{{\Delta }}}^{{\prime} }$. From left to right, we tune the relative critical temperature of ${{{\Delta }}}$ and ${{{\Delta }}}^{{\prime} }$. Time-reversal (TR) is broken in the blue region, whereas we have TRS in the pink region. **c**, Order parameters along four vertical cuts through the phase diagram. ${{{\Delta }}}^{{\prime} }$ always induces a subsidiary ${{{\Delta }}}$, while the converse is not true.

While in-plane strain can naturally produce an anisotropy by explicitly breaking rotation symmetry, the effect of an out-of-plane field on the transport anisotropy appears, at first glance, surprising. In the following, we elucidate the coupling of strain and magnetic field on a charge-density order parameter in kagome systems within Ginzburg–Landau (GL) theory. For this discussion, we focus on a single kagome layer and are guided by the following experimental observations: (1) there is translational symmetry breaking in the form of a 2 × 2 increase of the size of the (in-plane) unit cell; (2) there is no spontaneous rotational symmetry breaking in the absence of any external perturbation. This restriction will fix the terms of the GL expansion to fourth order in powers of the order parameter; and (3) the magnetic field couples linearly to the system, which restricts the form of the flux phase. We introduce a complex three-component order parameter ${\mathbf{{\uppsi }}}={{{\Delta }}}+i{{{{\Delta }}}}^{{\prime} }$ defined in Fig. 2a (and transformation properties defined in Supplementary Table 1). ${{{\Delta }}}$ describes charge bond order while ${{{\Delta }}}^{{\prime} }$ describes flux order and therefore breaks time-reversal symmetry. Importantly, this flux order is even under C2 symmetry, which is crucial for observation (3). The resulting GL free energy, shown in Methods, is extremely rich^51^ and includes not only third-order coupling^19,52–55^, but also a linear coupling of ${{{\Delta }}}$ with ${{{\Delta }}}^{{\prime} }$ mediated by the out-of-plane field^56^. In the absence of a magnetic field and strain, this free energy leads to the phase diagram shown in Fig. 2b, where from left to right we tune the relative critical temperature of the bond and flux order. As shown in the cuts in Fig. 2c, due to the presence of a third-order term in the free energy, any ${{{\Delta }}}^{{\prime} }$ induces a subsidiary ${{{\Delta }}}$, while the converse is not true. Finally, the third-order terms coupling the two order parameters result in an anisotropic solution. Given our observation (2), namely an isotropic order in the absence of external perturbations, we will focus in the following on the scenario 4 of the phase diagram presented in Fig. 2b.

## Field dependence of anisotropy and phase diagram

The above symmetry considerations on the Ginzburg–Landau level provide a field-strain-temperature phase diagram which reproduces the experimental observations remarkably well. The bond order ${{{\Delta }}}$ appears below *T*_CDW_ at a (weakly) first-order transition due to the presence of the third-order term. In the absence of a magnetic field or strain, there is no anisotropy, in other words the three components of ${{{\Delta }}}$ are identical. When a magnetic field is applied, ${{{\Delta }}}^{{\prime} }$ is induced leading to a non-zero anisotropy due to the third-order coupling of ${{{\Delta }}}$ and ${{{\Delta }}}^{{\prime} }$. This anisotropy becomes observable at a temperature below the charge-ordering temperature, when the third-order term becomes large enough. The anisotropy is further enhanced by increasing the magnetic field, since that increases the ${{{\Delta }}}^{{\prime} }$ component. In the presence of strain, an anisotropy in ${{{\Delta }}}$ is induced immediately at the charge-ordering temperature. Furthermore, at a lower temperature ${{{\Delta }}}^{{\prime} }$ can condense as well in the presence of strain, which increases the anisotropy. In both cases, TRS breaking only occurs when ${{{\Delta }}}^{{\prime} }$ is present.

**Fig. 3:**
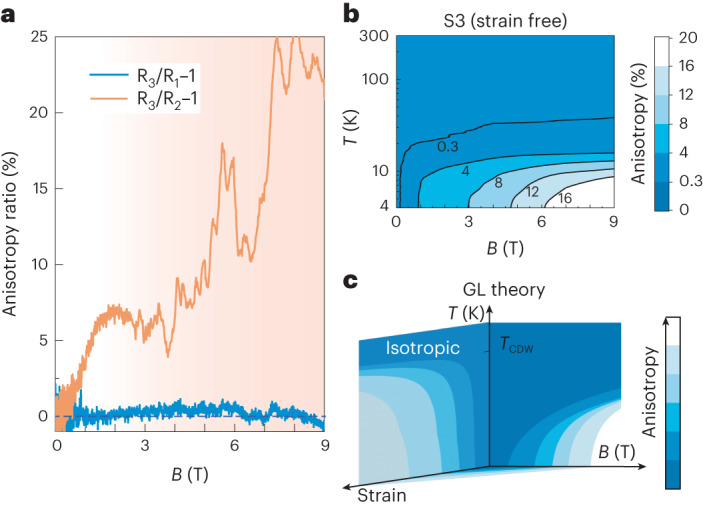
Anisotropic magnetoresistance and characteristic magnetic field scale. **a**, Field dependence of transport anisotropy in CsV_3_Sb_5_. **b**, Temperature-field phase diagram of anisotropy. The anisotropy stays zero without applying external magnetic field (*B* = 0 T) and above *T*' ≈ 30 K. **c**, Temperature-field-strain phase diagram reproduced based on Ginzburg–Landau theory, which is consistent with the experimental results displayed in **b**. Source data

In our scenario, pristine CsV_3_Sb_5_ is thus time-reversal symmetric at any temperature, yet located critically close to the TRS broken phase ${{{\Delta }}}+i{{{{\Delta }}}}^{{\prime} }$ in the phase diagram (Fig. 3). Applying the magnetic field directly promotes the coupling between the orders and drives an in-plane symmetry breaking at arbitrary low fields, that is without a threshold field. This scenario indeed matches the experimental data: Starting at zero in the absence of field or strain, the anisotropy immediately appears under field and continuously increases up to *B* = 2 T. At higher magnetic field, the anisotropy continues to grow yet at a slower pace and exhibits pronounced quantum oscillations. While microscopic details such as Landau quantization are naturally not considered in a GL theory, it is remarkable that its general trend continues despite the highly complex transport situation in the microstructure.

Finally, we note that raising the temperature pushes the system towards an isotropic state, which can be straightforwardly mapped out in our experiment (Fig. 3b). The thin isotropic sliver immediately gives rise to an anisotropic state under weakest magnetic fields, which is remarkably similar to the predictions of the GL theory shown in Fig. 3c. This behaviour can be directly rationalized from the phase diagram in Fig. 2b. In such a picture, raising the temperature pushes the system deeper into the isotropic, time-reversal symmetric state.

## Discussion and outlook

**Fig. 4:**
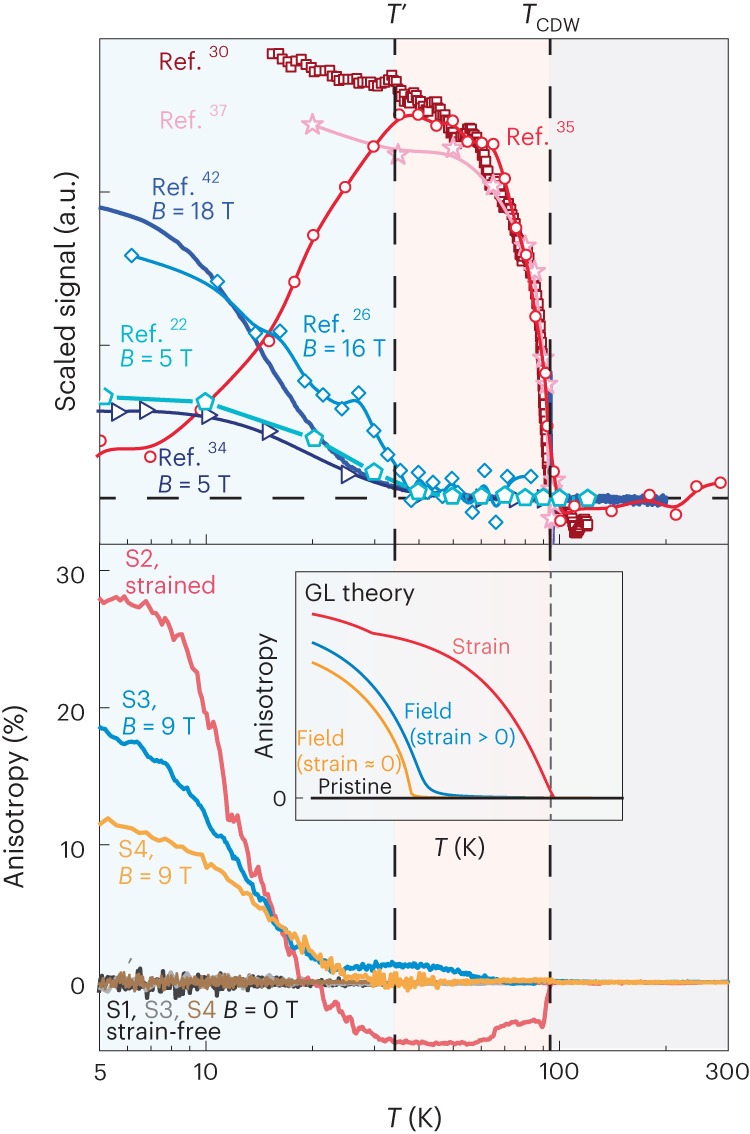
Summary and comparison with previous reports. Top, summary of the previously reported temperature dependence of anisotropy measured via various methods, including angle-dependent magnetoresistance^22,34^, electric magneto-chiral anisotropy^42^, elasto-resistivity^35^, nuclear magnetic resonance^26^, magneto-optical Kerr effect^30^ and optical polarization rotation measurement^37^. Interestingly, all results measured without magnetic field demonstrate that the anisotropy occurs once the charge order is formed. Meanwhile the in-field measurements consistently reveal yet another temperature scale (*T*') below *T*_CDW_. Bottom, the anisotropy measured in devices S1, S2, S3 and S4. The field-induced anisotropy occurs only below *T*_CDW_ and displays a gradual increase starts at *T*', while the strain-induced anisotropy onsets exactly at *T*_CDW_. The inset panel presents the *T*-dependent anisotropy of the order parameter under different conditions reproduced by Ginzburg–Landau theory. We choose Ginzburg–Landau parameters corresponding to scenario ④ in Fig. 2b,c. Source data

There have been numerous reports on the spontaneous symmetry breaking in CsV_3_Sb_5_ (Fig. 4). Despite the variety of experimental methods, most of the results recognize a six-fold to two-fold in-plane symmetry lowering upon entering the charge-ordered state. In contrast, our measurements of the nearly strain-free samples demonstrate that the electronic transport remains isotropic across the whole temperature range. We argue that this apparent discrepancy is mainly due to the fact that in most experiments, such as angular dependence of magnetoresistance and elasto-transport measurements, magnetic fields and uniaxial strain act as necessary probes of symmetry breaking/lowering. The extreme sensitivity to perturbations amplifies even accidental and in-adverted perturbations that may arise from crystal growth, handling or mounting in the experimental reality. Here, we have clearly demonstrated that these presumably small perturbations act as crucial tuning parameters of the transport anisotropy (Fig. 4). Importantly, there exist two critical temperature scales. Except for the apparent transition temperature *T*_CDW_ at which the strain-induced anisotropy appears exactly, the additional temperature scale *T*’ stands for the onset of the field-induced anisotropy. This is consistently demonstrated not only by our measurements but also all experiments with external magnetic field or uniaxial strain. These two distinct temperature scales suggest that despite the similar effect between strain and field, the way they couple to the order is different.

Our Ginzburg–Landau discussion provides an intriguing scenario that may reconcile some of the apparent contradictions reported in this field. In this picture, CsV_3_Sb_5_ exhibits an extreme sensitivity of the transport anisotropy due to its critical proximity to the loop-current phase. The anisotropy in the order parameters for cut 4 through the phase diagram in Fig. 2b reproduces successfully the influence of a magnetic field and strain on the temperature dependence of the anisotropy. Hence, our experiments, as well as the published literature, are consistent with a regime where the time-reversal-preserving bond order is dominant, while the TRS-breaking flux order is subdominant and is only induced in the perturbed case.

Our work once more highlights the rich responses and possible advanced functionalities in materials hosting a subtle balance of entangled orders, which can mutually be manipulated. The electronic state within the charge-ordered phase can be readily manipulated by weak strain and magnetic fields. Combined with the recently demonstrated chiral transport^42^ and optically manipulated chirality^43^, field-, strain- and light-tuned electric diodes become possible, albeit new materials with energy scales above room temperature are necessary for real applications. From a broader perspective, this work sends a beacon of hope into controversial fields which similarly may be unified when previously uncontrolled tuning parameters become controllable in the future, no matter how negligible and irrelevant they may seem at first glance.

## Methods

### Crystal synthesis and characterization

CsV_3_Sb_5_ crystallizes in the hexagonal structure with the P6/mmm space group. It contains layers of kagome planes formed by the V atoms. Following a self-flux procedure described in ref. ^17^, we obtained plate-like single crystals with typical dimensions of 2 × 2 × 0.04 mm^3^.

**Extended Data Fig. 1:**
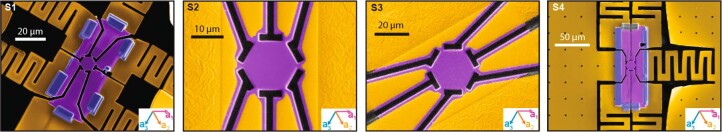
Scanning electron microscope (SEM) images of device S1, S2, S3 and S4. S1 and S4 are membrane devices with reduced differential thermal contraction strain, while S2 is glued down to a sapphire substrate, the coupling between the substrate and sample results in the non-negligible uni-axial strain at low temperature. S3 is a similar device with S2, yet after taking the SEM image, a glue droplet is applied on top of the device to reduce uniaxial strain at low temperature.

The micro-devices S1, S2, S3 and S4 are fabricated using the focused-ion-beam (FIB) technique (Extended Data Fig. 1). First, three slabs called lamellae are obtained from the same piece of bulk sample, and the plane of each lamella is aligned accurately to the kagome plane with less than ± 0.5° misalignment. For device S1, the lamella is transferred in-situ by a micro-manipulator and welded to a gold-coated (Au:100 nm) SiN_*x*_ membrane chip via Pt-deposition. The membrane window is about 200 by 200 μm, and its thickness is 100 nm. Soft meander-shaped springs are later fabricated to relax the thermal contraction strain with a typical spring constant ~100 N m^−1^. Meanwhile for both S2 and S3 the lamellae are transferred ex-situ and glued down to a sapphire substrate with prepatterned gold electrodes. All devices are exposed to a low-voltage (2 kV) Xe ion beam cleaning as the final fabrication step to reduce the thickness of FIB-damaged amorphous layer. After the fabrication of device S3, a small glue droplet of red araldite is added on top of the device via a thin wooden tip to reduce and homogenize the uniaxial strain observed in S2. The added glue thickness is about 30 μm. Since the thermal expansion coefficient of the glue is substantially larger than the device, it results in a compressive strain across the device and therefore compensates the tensile differential strain at low temperature. Furthermore, to demonstrate the reproducibility of the experimental results, another membrane-based device S4 was fabricated on a lamella cut from a different CsV_3_Sb_5_ crystal. Despite the slight difference in the exact temperature dependence of resistivity, the isotropic in-plane transport is consistently revealed (Fig. 1d).

**Extended Data Fig. 2:**
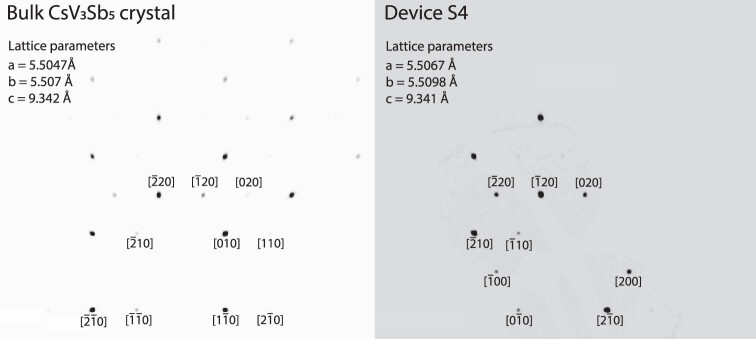
X-ray diffraction pattern of bulk sample and device S4. To check the crystalline alignment of the microstructure, we have performed X-ray diffraction measurements on both the bulk single crystal before FIB-fabrication (left) and the micro-structured device (right). The comparison demonstrates no change of crystalline structure. Note that as the substrate around the microstructure becomes a shadow for X-ray at certain angle range, the numbers of diffraction peaks are slightly reduced. The full angle X-ray measurement is accompanied by a video taken by the optical camera which records the motion of rotation in one degree step. We found that the crystallographic c-axis is aligned with the surface norm of the silicon substrate, demonstrating nearly zero misalignment. Based on the accuracy of such determination, the misalignment of crystalline axis is restricted to ± 0.5 degree.

To ensure that no apparent misalignment or change of crystalline structure is induced during the fabrication process, we have performed single crystal X-ray diffraction measurements of the bulk sample before FIB fabrication and the device S4 after the resistance measurements (Extended Data Fig. 2). This helps us to minimize the misalignment of crystalline axis down to less than 0.5°. The detailed comparison of crystalline lattice parameters demonstrates the unchanged crystalline structure after the fabrication process. Note that the small silicon opening window of the membrane device S4 limits the angle range of X-ray detection, rendering its Laue indexes weaker than the bulk sample.

**Extended Data Fig. 3:**
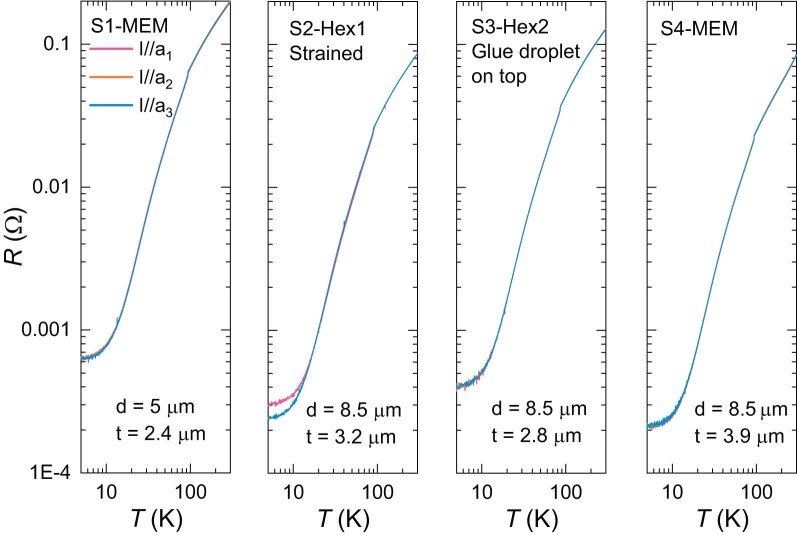
Temperature-dependent resistance of all four different devices. d and t stand for the edge length and thickness of the hexagon respectively. For S1, S3 and S4, the T-dependence of resistance measured along different axis is nearly identical with each other. This demonstrates the isotropic in-plane transport in these two devices. Meanwhile, for sample S2, the resistance measured with current along a_1_ displays a clear difference with the results measured along other current directions. This difference stands for the electronic anisotropy which increases with decreasing temperature.

Resistance measurements were performed using the resistivity measurement option in a Dynacool PPMS system with a maximal magnetic field of 9 T and base temperature of 2 K. The configurations of in-plane resistance measurements are illustrated in Fig. 1. For each current configuration, the voltages were measured along both sides of the device to ensure the current homogeneity. For all measurements a low AC current of 100 μA is used to avoid Joule heating. All temperature-dependence measurements were performed at a low sweeping rate of 1 K min^−1^. For all devices, the resistance varies from 0.2 Ω to 0.2 mΩ with a high residual resistivity ratio (RRR) above 300 (Extended Data Fig. 3). The high RRR and the clear observation of Shubnikov-de Haas oscillations demonstrate the unchanged crystalline quality of all devices after FIB fabrication as well as the irrelevance of FIB-damaged amorphous layer in the measurements of highly conductive materials.

To exclude the role of in-plane fields and misalignment of the magnetic field, full two-axis rotations were achieved via a standard PPMS rotator probe: one angle *θ* is set by a stepper motor while the angle *∅* is controlled by manual positioning outside of the cryostat. The exact angle is determined using the image taken under an optical microscope.

### Detailed analysis of strain effect

**Extended Data Fig. 4:**
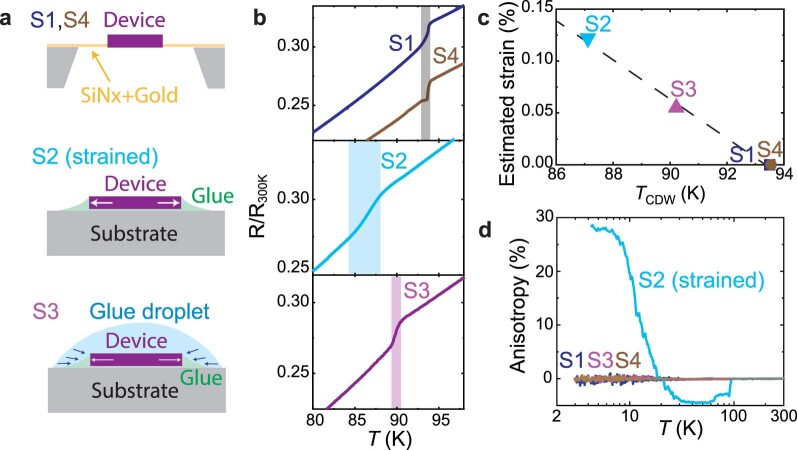
Strain analysis and T-dependence of transport anisotropy. (a) displays the detailed setup of each devices. The uniaxial strain due to differential thermal contraction is suppressed by the soft membrane spring in device S1, S4 and compensation of glue droplet in device S3. (b) Temperature dependence of resistivity near the charge density wave transition. Due to strain inhomogeneity, the transition is shifted to lower temperatures and becomes broader. (c) Estimating tensile strains of different devices based on the measured charge ordering temperatures. (d) Temperature dependence of transport anisotropy of all devices. S1, S3 and S4 display negligible anisotropy down to low temperature, while the strained sample S2 shows clear anisotropy which appears at the charge-density-wave transition and gets more pronounced at base temperature.

Each device displays a strain profile due to the different geometry. Devices S1 and S4 are membrane-based devices with micro-springs fabricated by FIB. These soft springs strongly compensate the thermal contraction strain which therefore results in a nearly strain-free situation (Extended Data Fig. 4). On the contrary, device S2 is directly attached to the sapphire substrate with glue. The mismatch of thermal contraction coefficient between CsV_3_Sb_5_ and sapphire results in a significant uniaxial strain. The uniaxial strain strongly influences the transport anisotropy of the device. Device S3 features the same setup with S2 yet a glue droplet is applied onto the top after the fabrication process. The larger thermal contraction coefficient of the glue results in a partially compensated and homogenized strain pattern. As a consequence, only the device S2 displays clear anisotropy after the charge order is formed while both S1 and S3 display no anisotropy at zero field down to low temperature. The thermal differential strain of each microstructure can be consistently checked by the charge-ordering temperature. The membrane-mount devices S1 and S4 display identical *T*_CDW_ as expected, while an apparent change of *T*_CDW_ for device S2 demonstrates the presence of tensile strain across the device. According to previous reports^48^, the estimated effective strain is around 0.12%. The broadening of the transition temperature also indicates an inhomogeneous strain distribution. The visible shift of *T*_CDW_ in device S3 is partially attributed to the homogenized pressure effect because of the glue droplet applied, causing an overestimation of tensile strain value displayed in Extended Data Fig. 4c.

**Extended Data Fig. 5:**
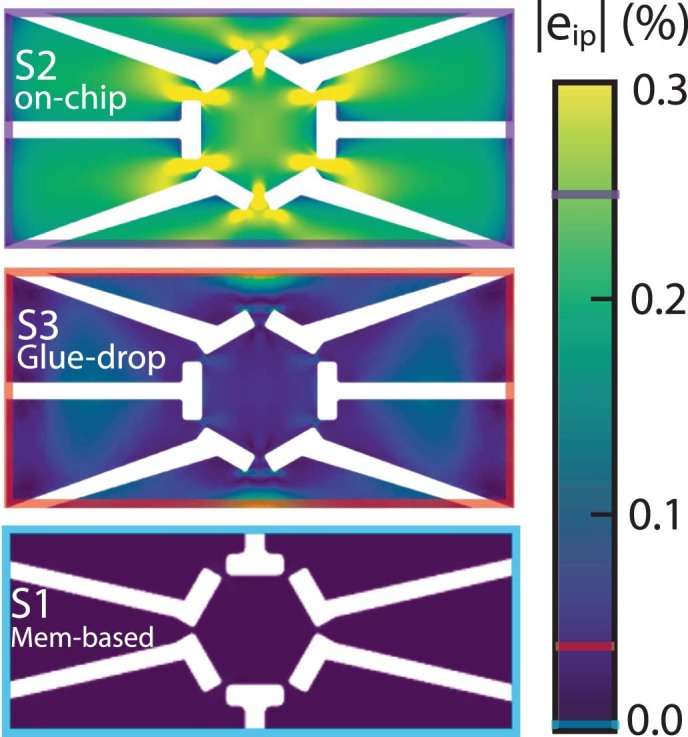
COMSOL simulation of uniaxial strain. Finite element simulation of strain distribution of device S1, S2 and S3 based on COMSOL multiphysics. The results display a dominant uniaxial strain component along the long side of the lamella.

To further demonstrate the strain profile difference, we also performed a finite element simulation using COMSOL multiphysics software (Extended Data Fig. 5). For the on-chip device S2, a strong and highly inhomogeneous in-plan strain component is obtained and the average value across the middle hexagon is estimated to be ~0.25%. With the application of the glue droplet, the in-plane strain is clearly suppressed, and the averaged value is only ~0.04%. Most importantly, the simulation results of the membrane-based device demonstrate a significant in-plane strain suppression as the averaged value is reduced to less than 0.002%, which is two orders of magnitude smaller than device S2. These results further demonstrate a modulation of in-plane strain profile due to the different device geometry.

### Temperature dependence of transport anisotropy and magnetoresistance

**Extended Data Fig. 6:**
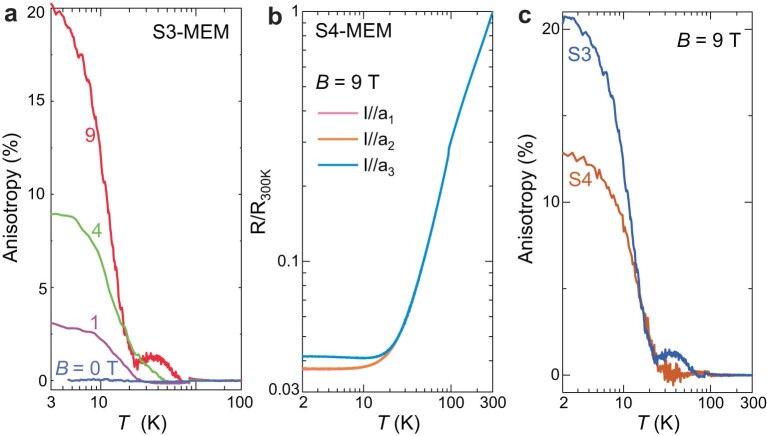
Field-induced transport anisotropy of device S3 and S4. (a) *T*-dependent transport anisotropy of S3 measured at different magnetic fields. The low temperature anisotropy increases strongly with increasing magnetic field. (b) *T*-dependent resistivity of S4 measured at different current directions with an external magnetic field of 9 T. (c) Transport anisotropy at 9 T for both S3 and S4. The difference is likely due to the difference in uniaxial strain across the device.

The temperature dependence of the in-plane anisotropy under various magnetic fields (Extended Data Fig. 6) demonstrates its strengthening in field. Not only the absolute value increases, also the onset at which the anisotropy becomes visible increases from 25 K at *B* = 1 T to 70 K at *B* = 9 T. Note that this temperature dependence is slightly different from the uniaxial strain case as the anisotropy never becomes fully negative. Compared to S3, device S4 displays a smaller anisotropy at low temperature, most probably due to the difference in strain distribution across the device (Extended Data Fig. 6). This demonstrates a direct coupling between the magnetic field and uniaxial strain, consistent with the prediction by the Ginzburg–Landau theory.

**Extended Data Fig. 7:**
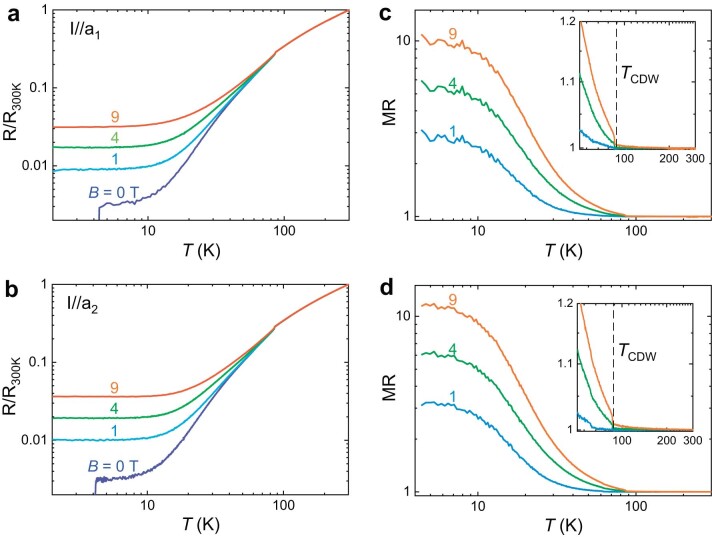
T-dependence of magnetoresistance for S3. (a) and (b) display the T-dependence of resistance measured at various magnetic fields with current along a_1_ and a_2_ respectively. The magentoresistance ratio [*R*(*B*)/*R*_0_] for both current directions are presented in (c) and (d) respectively. It is clear that MR only becomes significant below the charge-ordering temperature.

The temperature dependence of the magnetoresistance (MR), [*R*(*B*)/*R*(0)], is consistent with previous report as it only becomes significant below *T*_CDW_(Extended Data Fig. 7)^45^. This indicates the Fermi surface reconstruction due to charge ordering, which leads to the appearance of three-dimensional Fermi pockets and consequently the visible magnetoresistance.

**Extended Data Fig. 8:**
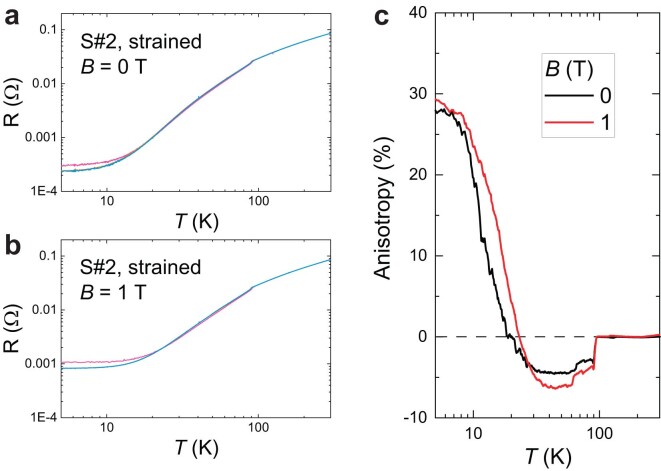
T-dependent resistance and in-plane anisotropy of S2 at *B* = 0 and 1 T. (a) and (b) present *T*-dependence of directional resistance measured at zero field and *B* = 1 T. The anisotropy due to strain is slightly enhanced by magnetic field, as shown in (c).

This trend continues when both magnetic field and strains are applied simultaneously. The transport anisotropy of device S2 increases when an external magnetic field of *B* = 1 T is applied (Extended Data Fig. 8).

### Angular dependence of magnetoresistance

**Extended Data Fig. 9:**
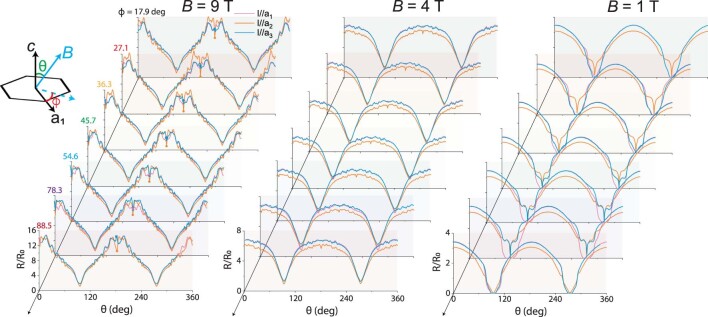
Angular dependence of MR (AMR) for S3. AMR measured with spherical rotation and different magnetic fields. The difference between *I*//a_2_ and other two current directions can be clearly seen for all *∅*, demonstrating the intrinsic nature of such behavior.

In magnetotransport experiments, field misalignment is a common extrinsic source of lowered apparent symmetry. In our case, to examine the effect of field misalignment away from the c direction on the anisotropic transport we report, an extensive angular dependence of MR measurements have been performed with not one but two rotational axes to cover all possible field directions in three-dimensional space (Extended Data Fig. 9). The rotation from out-of-plane to in-plane direction with the angle *θ* is controlled by the rotator motor on the PPMS horizontal rotator with a 0.5° per step resolution. Meanwhile, the in-plane rotation is done by manually altering the position of the device on the rotator puck. For each measurement a high-resolution image is taken via the optical microscope, and the angle *∅* is determined digitally using the alignment marker on the image. With external field *B* = 9 T, except for the clear dip around *θ* ≈ 180°, note that at smaller *∅*, the MR measured with *I*//**a**_2_ is larger than the MR for other two current directions. The difference is gradually suppressed and becomes invisible for *∅* larger than 46°.

This behaviour is closely related to the admixture of low longitudinal MR set by the angle between the magnetic field and current directions, which explains the variation of MR difference with in-plane rotation. The dip at *θ* ≈ 180°, however, is insensitive to in-plane rotation, as it can be consistently observed in all *∅* angles. This demonstrates an intrinsically lower magnetoresistance for *I*//**a**_2_ with *B*//*c*, which is insensitive to misalignment between the sample and magnetic field. In particular, it is not tuned by the residual in-plane magnetic field component. This point can be further elaborated by the results measured at a lower magnetic field (*B* = 1 and 4 T) as a clear difference between *I*//**a**_2_ and other directions is observed over a wide range of angle *θ*. This is mainly due to the reduced effect of the longitudinal MR component and smaller quantum oscillation amplitude at a lower magnetic field. Thus we conclude that the observed in-plane anisotropy is an intrinsic property of CsV_3_Sb_5_ which is robust against extrinsic perturbations such as misalignment.

### Field dependence of magnetoresistance

**Extended Data Fig. 10:**
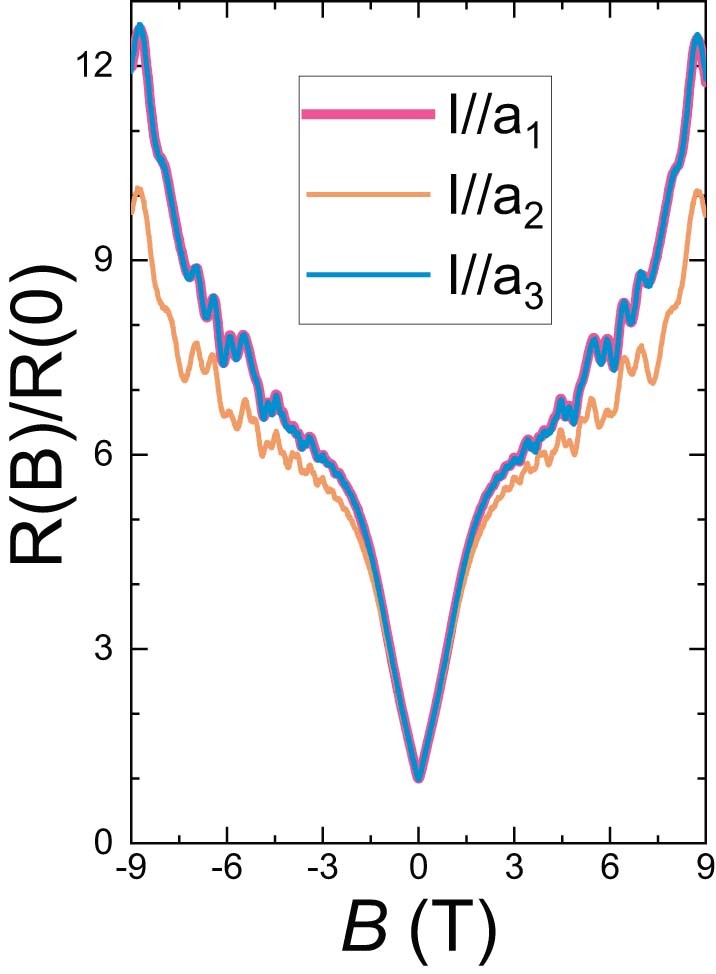
Magnetoresistance along different directions. Field dependence of magnetoresistance measured along all three different current directions at *T* = 2 K in device S3. The results measured with *I*//a_1_ and *I*//a_3_ overlaps exactly with each other, yet deviates clearly with *I*//a_2_. All magnetoresistances display a shoulder-like feature at around *B* ≈ 1.6 T.

The magnetoresistance has been measured along all three different current directions. The resistance for *I*//**a**_2_ displays a clear difference compared to the other two curves, from which the anisotropy displayed in Fig. 3a was extracted. Meanwhile all curves display a shoulder-like feature at *B* ≈ 1.6 T (Extended Data Fig. 10), consistent with previous reports^45^. This feature corresponds well with a plateau in the field dependence of transport anisotropy (Fig. 3a).

## Online content

Any methods, additional references, Nature Portfolio reporting summaries, source data, extended data, supplementary information, acknowledgements, peer review information; details of author contributions and competing interests; and statements of data and code availability are available at 10.1038/s41567-023-02374-z.

### Supplementary information

Supplementary Information Supplementary Fig. 1, Discussion and Tables 1–2.

### Source data

Source Data Fig. 1 Raw data for resistivity measurements.

Source Data Fig. 3 Data for scaled magneto-anisotropy.

Source Data Fig. 4 (a) S1 to S4, data for the temperature dependence of resistivity anisotropy. (b) Theory, data for theoretically predicted anisotropy versus scaled temperature.

## Data Availability

Data that support the findings of this study are available at Zenodo with the access link: 10.5281/zenodo.10076422. Source data are provided with this paper.

## Code availability

Code that support the findings of this study are available at Zenodo with the access link: 10.5281/zenodo.10076422.
