# Supplementary material for: Correlated order at the tipping point in the kagome metal CsV3Sb5
Source: Nat Phys. 2024 Jan 31;20(4):579–84. doi: 10.1038/s41567-023-02374-z (PMC11021193; doi:10.1038/s41567-023-02374-z)
Supplement: Supplementary file 1 — Supplementary Fig. 1, Discussion and Tables 1–2. [file 41567_2023_2374_MOESM1_ESM.pdf]

nature physics

Article

https://doi.org/10.1038/s41567-023-02374-z

Correlated order at the tipping point in the kagome metal CsV_3Sb_5

In the format provided by the authors and unedited

Ginzburg-Landau theory

We consider the order parameters Δ_i and Δ'_i as defined in Fig. 2a, which have the transformation properties listed in Tab. I.

TABLE I. Transformation properties of the order parameters Δ_i and Δ'_i, where t_i are translations by a lattice vector, C_n are rotations and σ_{vi} are three mirror planes.

	t_1	t_2	t_3	C_6	C_3	C_2	σ_{v1}	σ_{v2}	σ_{v3}
Δ_1	Δ_1	$-\Delta_1$	$-\Delta_1$	Δ_3	Δ_2	Δ_1	Δ_1	Δ_3	Δ_2
Δ_2	$-\Delta_2$	Δ_2	$-\Delta_2$	Δ_1	Δ_3	Δ_2	Δ_3	Δ_2	Δ_1
Δ_3	$-\Delta_3$	$-\Delta_3$	Δ_3	Δ_2	Δ_1	Δ_3	Δ_2	Δ_1	Δ_3
Δ'_1	Δ'_1	$-\Delta'_1$	$-\Delta'_1$	Δ'_3	Δ'_2	Δ'_1	$-\Delta'_1$	$-\Delta'_3$	$-\Delta'_2$
Δ'_2	$-\Delta'_2$	Δ'_2	$-\Delta'_2$	Δ'_1	Δ'_3	Δ'_2	$-\Delta'_3$	$-\Delta'_2$	$-\Delta'_1$
Δ'_3	$-\Delta'_3$	$-\Delta'_3$	Δ'_3	Δ'_2	Δ'_1	Δ'_3	$-\Delta'_2$	$-\Delta'_1$	$-\Delta'_3$

The general (Ginzburg-Landau) free energy including coupling to an out-of-plane magnetic field B_c and strain in the form of a strain tensor ϵ_{ij} is[1–4]

$$\begin{aligned}\mathcal{F}[\vec{\Delta},\vec{\Delta}'] =& \alpha(T-T_\mathrm{c})(\Delta_1^2+\Delta_2^2+\Delta_3^2)+\alpha'(T-T'_\mathrm{c})(\Delta_1'^2+\Delta_2'^2+\Delta_3'^2)\\ &+\beta_1\Delta_1\Delta_2\Delta_3+\beta_2(\Delta_1\Delta'_2\Delta'_3+\Delta'_1\Delta_2\Delta'_3+\Delta'_1\Delta'_2\Delta_3)\\ &+\mu_1 B_c(\Delta_1\Delta'_1+\Delta_2\Delta'_2+\Delta_3\Delta'_3)\\ &+\mu_2[(\epsilon_{xx}-\epsilon_{yy})(\Delta_1^2-\Delta_2^2/2-\Delta_3^2/2)+\epsilon_{xy}\sqrt{3}(\Delta_2^2-\Delta_3^2)]\\ &+\mu_3[(\epsilon_{xx}-\epsilon_{yy})(\Delta_1'^2-\Delta_2'^2/2-\Delta_3'^2/2)+\epsilon_{xy}\sqrt{3}(\Delta_2'^2-\Delta_3'^2)],\end{aligned} \tag{S1}$$

where we have dropped fourth-order terms for simplicity. For the calculation of the full phase diagram, these are chosen in such a way that the perturbation-free case yields an isotropic solution, where we define the anisotropy as $\sum_{i\neq j}(\Delta_i^2-\Delta_j^2)^2+\sum_{i\neq j}(\Delta_i'^2-\Delta_j'^2)^2$. Let us note three important aspects of this free energy: (1) The anisotropy can be induced either through the third-order terms proportional to β_1 and β_2 (the latter of which is only active when both order parameters are present) or due to the explicit strain coupling proportional to μ_2 and μ_3. (2) $\vec{\Delta}'$ always induces a subsidiary $\vec{\Delta}$ due to the third-order term proportional to β_2, but not the other way around. (3) in the presence of a magnetic field, $\vec{\Delta}$ and $\vec{\Delta}'$ are always coupled linearly and therefore appear together.

Taking (1) and (2) together explains the (perturbation-free) phase diagram in Fig. 2b, which shows the different scenarios as a function of $T_\mathrm{c}-T'_\mathrm{c}$. For $T'_\mathrm{c}<T_\mathrm{c}$, $\vec{\Delta}$ and $\vec{\Delta}'$ always appear

TABLE II. Choice of Ginzburg-Landau coefficients used for Fig. 3c and Fig. 4 inset.

Parameter	α	α'	β_1	β_2	μ_1	μ_2	μ_3
Value	1	1	1	-1	1	1	0.5

together due to the β_2 term, such that time-reversal symmetry is broken. $\vec{\Delta}$ is much smaller than $\vec{\Delta}'$, since it is induced by the latter. For lower temperatures, or when $T_c' \sim T_c$, $\vec{\Delta}$ increases and we cross over to a regime where both order parameters have a comparable magnitude. Finally, when $T_c > T_c'$ (and in the absence of strain and magnetic field), $\vec{\Delta}$ but not $\vec{\Delta}'$ is non-zero at the charge-ordering temperature and time-reversal symmetry is preserved.

The inset of Fig. 4 is obtained by numerically minimizing the free energy for a representative set of the coefficients (Tab. II), corresponding to scenario (4) in Fig. 2b and computing the anisotropy as a function of T. While there is no anisotropy in the pristine case (without strain or B_c), applying a magnetic field induces $\vec{\Delta}'$, which in turn leads to anisotropy in the order parameter due to the β_2 term. Finally, strain trivially leads to an anisotropy via the μ_2 term.

In Supplementary Fig. S1 we show the results for the anisotropy from the GL theory for a different set of GL parameters corresponding to regime (1), where the TRS-breaking order is dominant. This regime is inconsistent with experimental observations, since it shows a suppression of the anisotropy when a magnetic field is applied.

Not captured by our two-dimensional theory is the possibility that an isotropic response of the pristine system is the result of averaging over many layers with randomly distributed anisotropies (i.e., domains in the stacking direction). However, we consider this possibility unlikely for the following reason. Upon applying the magnetic field, the layers would need to align their anisotropies to yield the experimentally observed macroscopic anisotropy. In contrast, our GL theory would imply that each layer becomes more anisotropic in the magnetic field. The random stacking would then still result in an averaging to an isotropic response.

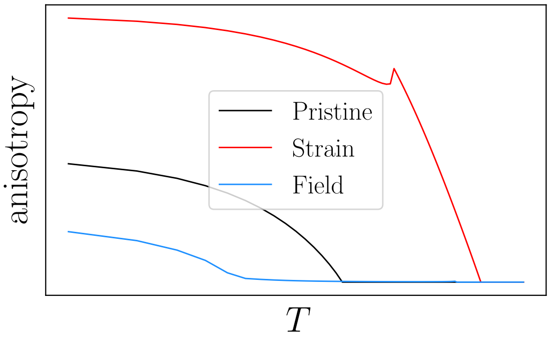

Fig. S 1. **Anisotropy from GL theory in regime ①.** The pristine, strained and magnetic field cases all show an anisotropy and in particular the magnetic field suppresses the anisotropy contrary to the experimental observations. The anisotropy in regime ① is therefore not consistent with experiments.

REFERENCES

[1] Denner, M. M., Thomale, R. & Neupert, T. Analysis of Charge Order in the Kagome Metal AV_3Sb_5 (A = K, Rb, Cs). Phys. Rev. Lett. **127**, 217601 (2021).

[2] Grandi, F., Consiglio, A., Sentef, M. A., Thomale, R. & Kennes, D. M. Theory of nematic charge orders in kagome metals. arXiv:2302.01615 (2023).

[3] Tazai, R., Yamakawa, Y. & Kontani, H. Drastic magnetic-field-induced chiral current order and emergent current-bond-field interplay in kagome metal AV_3Sb_5 (A=Cs,Rb,K). arXiv:2303.00623 (2023).

[4] Christensen, M. H., Birol, T., Andersen, B. M. & Fernandes, R. M. Loop currents in $A\mathrm{V_3Sb_5}$ kagome metals: Multipolar and toroidal magnetic orders. Phys. Rev. B **106**, 144504 (2022).
